# Counseling Techniques Supporting West African Children With Adverse Childhood Experiences: A Systematic Review

**DOI:** 10.3389/fsoc.2021.573115

**Published:** 2021-06-07

**Authors:** Shanelle V. Clay, Cheria Jackson, Quincy Stephenson

**Affiliations:** Department of Counseling, Psychology, and Special Education, Duquesne University, Pittsburgh, PA, United States

**Keywords:** Western Africa, counseling, children, intervention, techniques, counseling techniques, adverse childhood adversities

## Abstract

Using a meta-synthesis approach, through the review of current literature, five published and peer reviewed qualitative research reports were studied. The intention was to identify interventions being used with West African children who have endured adverse childhood experiences. These results were found through matching inclusionary criteria and all studies were screened for appropriateness and relevance to the topic matter. The literature was analyzed across five online databases including Proquest, PsychInfo, Scopus, Wiley, and Springer from January 2005 to June 2020. The authors found minimal evidence indicating interventions used in West Africa for adverse experiences related specifically to children, but found themes related to interventions that serve West African families that include children. Findings were thematically analyzed through meta-synthesis and identified four themes used in the interventions, which include western, spiritual, expressive arts, and cultural approaches. West African children endure adverse experiences such as terrorism, abuse, and war violence that contribute to an increasing the need for mental health interventions. These experiences approached from western, spiritual, expressive arts, and cultural vantage points were identified but limited in information about delivery and efficacy, thus providing little guidance regarding further exploratory research.

## Introduction

Adverse childhood experiences (ACES), can be described as distressing and traumatic involvements during youth and adolescence such as exposure to abuse (sexual, physical, mental, emotional), introduction to violence, household dysfunction, and separation of family (Kimple and Kansagra, 2018). According to a report conducted by CDC-Kaiser, adverse childhood experiences are common across the world with indication of some populations with a higher probability of exposure due to contrary social and economic circumstances (Felitti et al., 1998). The research steered to identifying the adverse childhood experiences on a global perspective, showing an impact on socio-emotional learning, cognition, and health status after exposure to trauma. Addressing the impact of mental health on school-aged children can be beneficial to their learning. Children exposed to adverse experiences are found to have a greater negative impact on health status, which may reduce their lifespan (Herzog and Schmahl, 2018).

West African children are exposed to adverse childhood experiences that are unique in comparison to other parts of the world. An article by Herzog and Schmahl (2018) states, malnutrition, exposure to violence through witnessing death of family members, committing murder or violent acts as child soldiers, sexual violence, genital mutilation, and war are listed as potential adverse childhood experiences in West Africa. In a presentation by Ogunkua et al. (2019), an ACES self-report was completed by teachers in training to reflect their own childhood trauma in Nigeria. The results showed that over 66% of the respondents who work in schools reported to have at least one adverse childhood experience and one in five of the responders reported having three or more experiences from a pool of 826 participants. The results highlight that West Africans are exposed to many experiences due to adverse issues such as war, sexual abuse, violence and emotional neglect (Ogunkua et al., 2019). Children who live through these experiences would benefit from counseling interventions to regulate socio-emotional learning and reduce other mental health issues, that can come from these traumatic experiences such as alcoholism, substance abuse, and other risky behaviors (Kabiru et al., 2010).

Counseling in indigenous communities, such as West Africa, requires the acknowledgment that modern practices are not easily accepted within the community (Levers et al., 2011). Articles that address counseling interventions for adverse childhood experiences in West Africa are limited. Previous education on traumatic experiences impacting Africa's indigenous people primarily focus on South Africa (Jewkes et al., 2010), with studies pooled from people in Capetown and Johannesburg (Felitti et al., 1998). This shortage of mental health data suggests that insight for this sub-group of people is needed; therefore, summoning the necessity for this article.

## Methodology

The primary role of a systematic review of the literature is to identify and examine the available research for specific research queries. Research is inspected using a protocol for retrieval and reliability which respond to the scope of the study. It is important to have knowledge of indigenous cultures when conducting research in another country. All of the researchers in this article identify as African American. The research team discussed cultural implications and potential differences in cultures to reduce bias.

The research questions should be identified before completing the search, as it impacts the entire process for the systematic review (Wheeler and Richards, 2007). The point of this qualitative study is to introduce a compact diagram of the current writing to respond to the lack of information on techniques and interventions used for West Africa's school-aged children with adverse childhood experiences. These interventions will include components highlighting the mental, physical, and spiritual component of wellness as it applies to the acknowledged population.

The systematic review conducted by the researchers in this analysis focuses literature from January 2005 to June 2020 related to adverse experiences and children. The purpose was to examine the adverse experiences that children in West Africa endure and to understand interventions that are used in therapeutic efforts to alleviate the effects of those experiences.

Adverse experiences can be defined as traumatic exposures to abuse, violence, dysfunction (Kimple and Kansagra, 2018). Using a three-phase methodology of a key term search, filtering by date relevance of January 2005 to June 2020, and content screen review, the researchers were able to gather data that highlighted adverse experiences and interventions that were either used in the literature or suggested for the West African population, including children, for addressing mental health symptoms.

Qualitative research has historically been chosen as the preferred research method to recount phenomenological perspectives, interpret behavior, and understand cross-cultural information (Lazaraton and Taylor, 2007; Rahman, 2017). Rather than quantitative investigations of viability, which plan to assess results regarding outcomes, a qualitative approach offers a more in-depth perspective from the participant. Qualitative research provides a subjective examination that does not rely on the researcher for data; therefore, using a subjective examination supports this study in identifying best practices for children and adolescents in West Africa. Our investigation about clinical practices used with West African people, provided minimal perspective and no formal model for treatment; therefore, a subjective examination into the perspective of treatment by children, adolescents, and families was completed. The information identified through qualitative review may foster implications for improved care. The key aim of this systematic analysis is to determine the efficacy of interventions for children in West Africa who have encountered adverse conditions during their childhood.

### Database Searches

There was a review of five computer databases: Proquest, PsychInfo, Scopus, Wiley, and Springer. These databases were searched online using a university sponsored search engine and can be found in Table 1 of this article. The database selection remained limited to these search database due to restriction in application. The searches included key terms to filter down the articles to keep the most relevant and appropriate articles for review. Beginning the search at African counseling techniques (interventions), West Africa, children, adverse experiences, qualitative, restricted dates for articles from January 2005 to June 2020 and excluded any results that were not from peer reviewed journals. In reviewing this time frame, we are able to see the influx on mental health articles over a range of time impacted by political changes and discovery in mental health. In applying this methodology there were challenges with initial searches due to lack of results in searching West Africa generally. To accurately search for articles that apply to West Africa, the researchers also searched the articles by each individual country that represents West Africa. These were identified by the Encyclopedia Britannica as the countries of Benin, Burkina Faso, Cameroon, Cabo Verde, Chad, Cote d'Ivoire, Guinea, The Gambia, Ghana, Guinea, Guinea-Bissau, Liberia, Mali, Mauritania, Niger, Nigeria, Senegal, Sierra Leone, and Togo (Britannica, 2019).

**Table 1:** Database search results.

	**Psychinfo (2005–2020)**	**Proquest (2005–2020)**	**Springer (2005–2020)**	**Wiley (2005–2020)**	**Scopus (2005–2020)**	**Total**
	Citations of key term per database					
African counseling techniques	413	318	2,542	23,781	1,606	28,978
Western Africa	5	97	2,542	1,838	19	4,498
Children	3	–	2,046	1,090	10	3149
Adverse experiences		–	675	570	–	1,245
Total	3	97	675	27,279	1635	29,689

## Results

**Figure 1:**
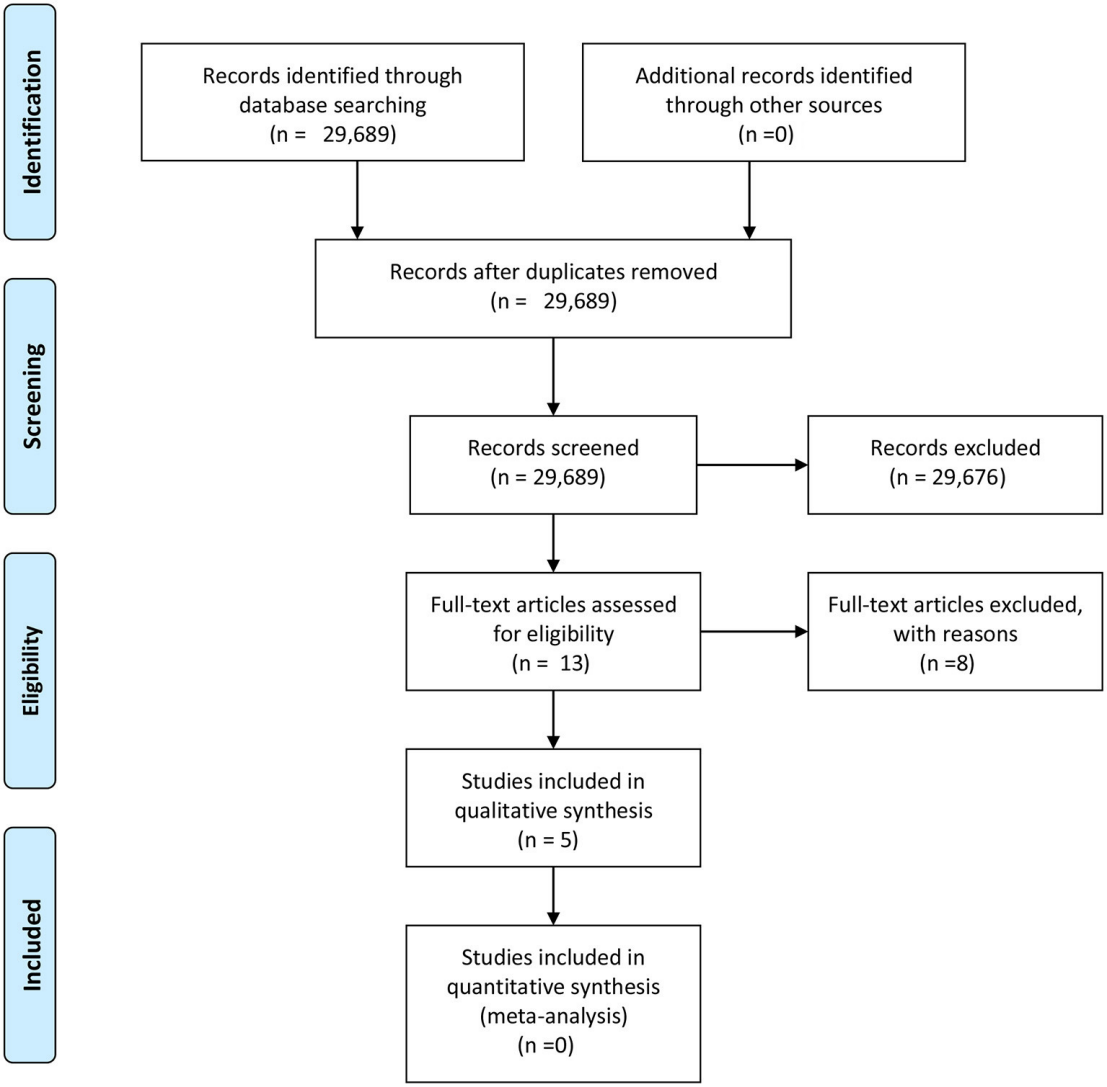
Prisma flow chart for journal findings (Moher et al., 2009).

The researchers restricted the search to qualitative studies and date ranges of January 2005 to June 2020, to support the systematic review needed to assess the current state of interventions related to adverse experiences with West Africa's children. Following this algorithm 29,689 articles were identified as presented in Figure 1 of the PRISMA flow diagram (Moher et al., 2009). Thirteen articles were assessed for eligibility in terms of focus on children and families with children, leaving eight articles to be removed. After filtering the articles for desired population, appropriateness, qualitative factors, removing abstracts, keeping articles focused on psychology and child and family studies, the result was five articles that were used to explain interventions. Descriptive characteristics of the articles are in Table 2. Following the article extraction and analysis it was found that the research suggests interventions of use clinically within the West African culture that focus on themes surrounding spirituality, culture, expressive arts, and Western approaches to psychotherapy.

**Table 2:** Five journal articles selected between 2005 and 2020 included in systematic review.

**Author(s)**	**Year**	**Title**	**Discipline**	**Journal**	**Interventions and Themes**
Lo and Dzokoto	2005	Talking to the master: Intersections of religion, culture, and counseling in Taiwan and Ghana	Clinical Mental Health	Journal of Mental Health Counseling	Western-style psychotherapy, public health education, religious mentoring, The Ideal Master Approach Themes: Western Psychotherapy, Spirituality, Culture
Stepakoff et al.	2006	Trauma Healing in Refugee Camps in Guinea: A Psychosocial Program for Liberian and Sierra Leonean Survivors of Torture and War	Psychology	The American Psychologist	Stage-oriented model of trauma recovery (psychodynamic, relational/interpersonal, cognitive–behavioral, narrative, and expressive/humanistic psychotherapies), affect-laden experiences in words or other symbolic forms (e.g., drawings, drama, songs). Themes: Western Psychotherapy, Expressive Arts
Weine	2011	Developing preventive mental health interventions for refugee families in resettlement	Family Studies/Psychology	Family Process	Resilience approach, community collaboration, Mixed Methods With Epidemiology, and Focused Ethnography Preventative mental health interventions (Feasibility, Acceptability, Culturally Tailored, Multilevel, Time Focused, Prosaicness, Effectiveness, and Adaptability) Social Support, Peer Groups Themes: Western Psychotherapy
Asagba	2015	Developing logotherapeutic strategies as effective interventions for victims of sexual assault	Psychology	Gender and Behavior	Frankl mountain range, Long's logotherapeutic transcendent crisis intervention 3-step model as logohint or logohooks for managing of the sexual assault victims Themes: Expressive Arts
Ebigbo et al.	2017	Cross cutting issues in the practice of psychotherapy in Nigeria	Clinical Psychology	Journal of Contemporary Psychotherapy	Prayer houses, traditional healers, Harmony Restoration Therapy and Meseron treatment frameworks, cosmogram. Wawa-Technique, Culture Centered Psychotherapy Themes: Spirituality, Culture

### Spiritual

The introduction of modern counseling practices for indigenous communities requires cultural competency and knowledge of their world view. Traditional African culture emphasizes great respect for and wisdom in their community leaders such as village chiefs, elders, traditional healers, and ancestors. It is to them that one goes for guidance, direction, and atonement; therefore, these are their “counselors” (Levers et al., 2011). Although many of the interventions practiced by traditional healers may not be applicable to children, children and adolescents are influenced by these healers due to the nature and culture of their environment and influence on the family (Lo and Dzokoto, 2005). The extracted articles identify the importance of indigenous psychological practices from a spiritual and creative lens.

Two of the extracted articles indicated that spirituality was a preferred counseling intervention for children in West Africa who have had adverse childhood experiences. Ebigbo et al. (2017) qualitative study acknowledges the importance of spirits—use of traditional healers—and prayer houses as interventions for therapy. The spirits provide a sense of consciousness and guidance of morally accepted ideals. The prayer houses are a popular place to reduce stress and identify aspects of hope in times of adversity. Prayers serve as meditation and instant gratification because those who appear at these prayer houses serve as each other's support with the guidance of a priest. This act is like group counseling with a facilitator. Traditional healers are the preferred facilitators of these healing groups fostered in the prayer houses.

Ebigbo's Harmony Restoration Therapy acknowledges the importance of spirituality in the healing techniques of the Igbo people by identifying the need for mending and healing amongst one's own self and one's relationships with others (Ebigbo et al., 2017).

A qualitative study by Lo and Dzokoto (2005), recognized the importance of acknowledging spirituality and the balance of technique as it applies to counseling. This study provides implications of interventions used with Ghanaian clients with the counseling framework of the approach of the Ideal Master (IM). Approaches used by the Ideal Master identify ways to manage stress through reduction of anxiety, positive self-talk, and prayer, and are unified to eliminate cognitive distortion due to traumatic experiences, such as genital shrinking (Lo and Dzokoto, 2005).

### Culture

The foundation of African society includes the identity of the family and community.

People are viewed as a section of their natural setting and are closely meshed in the practices and beliefs through experience and education from family (Kamya, 1997). The incorporation of culture into the application of treatment interventions for West Africa's community members, including children, creates an experience that is unique to individuals within a regional area. As previously mentioned the Ideal Master not only uses spirituality but culture as well to facilitate interventions. The Ideal Master (IM) is a counselor who is knowledgeable and aware of cultural values, religious beliefs, and Western Counseling theories (Lo and Dzokoto, 2005). They are able to process the importance of culture and religion in the worldview of the client. IMs integrate all three previously mentioned factors to understand and encourage mental health wellness. IM is not easily achieved in practice, as incorporating cultural and spiritual factors with Western trained counselors may be a difficult task for more native clients (Lo and Dzokoto, 2005). Western trained counselors should be aware of the cultural and spiritual norms in the client's native community. In understanding the norms, this assists the counselor in processing problems and developing conceptualizations more effectively and appropriately. Counselors should also know how a client engages in cultural and spiritual practices as it applies to their life. Counselors should consult where appropriate to increase their cultural and religious knowledge (Lo and Dzokoto, 2005). Lastly, IM counselors should be considerate of the various social identities a client may have in avoiding generalization based upon one factor of their identity. Differences in history and personality as well as sexual orientation, physical ability, gender, socio-economic status, and age contribute to individuals' experiences and should be considerations in assessment and intervention development and application (Lo and Dzokoto, 2005).

Other interventions steeped in culture include culture centered psychotherapy in the form of Harmony Restoration Therapy, Meseron treatment frameworks, cosmogram, and the Wawa Technique (Ebigbo et al., 2017). Human Restoration Therapy (HRT), is based on African culture and used in Nigeria. It is the concept of belief in supernatural forces, expectations of supernatural punishment, belief in symbols, tribal legends and mythological concepts, affective activity in everyday life, identification with cultural group, decreased ego boundaries, ancestor worship, belief in dream life as a reality, belief in idealized good objects, use of symbols and grand religious belief (Ebigbo et al., 2017). HRT focuses on the relationship between humans, spirits, gods, and the Almighty God as a part of the sanity of Africans. It is a holistic view that incorporates ancestors, spirits and nature and focuses on the relationships that an individual has with these entities. If there is discourse in those relationships the wellness and health will not be well for the person until the relationships are mended (Ebigbo et al., 2017). Harmony Restoration Theory acknowledges that there is a mind, body, society, psychosocial, and environmental thread that is culture based and innate to West Africans, enabling them to reach beyond their individual selves.

Meseron Therapy is a psychotherapeutic method that is native to Nigeria based on the language and culture of Nigerians. Maseron equates to “I refuse.” It focuses on the assumptions that an individual's decisions hold consequences that are inevitable, as well as that individuals have the capacity to change their condition by working on themselves (Awaritefe, 1995). In Meseron treatment an individual detaches themselves from negativity and actively embraces and surrounds themselves with positive things (Ebigbo et al., 2017). In Meseron, the therapist assists the client in moving to a place that promotes the strength to refuse actions and be self-motivated and determined to overcome their illness. The goal is to encourage the awakening of the client to think about their situation, use mobilization of their resources to change their situation, and assist in symptom relief or termination of the unwanted condition of the client (Ebigbo et al., 2017).

Cosmograms are used in therapy to create interview maps of the areas of important relationships and to display the levels of harmony or disharmony within them (Ebigbo et al., 2017). With the purpose of restoration, a cosmogram maps out all the relationships that are important to the individual. Using detailed psychological exploration that focuses on the sources of belief systems and usual relationships in West Africa, such as immediate and extended family, a cosmogram illustrates the client's belief systems and any discourse within the relationships (Ebigbo et al., 2017). These are used to discover the functional and non-functional relationships and how they relate to each other, understand if there are any breaches of promises or oaths within the relationships as well as identifying successes and failures. Tangible components of the client's life that are of value are also explored. In evaluating these components of the client's life the client should have a fair understanding of the relationships that interact with their life and encourage the family to process their relationships (Ebigbo and Onuora, 1987). This is usually observed by a Harmony Restoration Therapist to protect from superstitious, exploitative religious and traditional healers (Ebigbo et al., 2017). The Harmony Restoration Measurement Scale is used as an objective psychometric measure of the cosmos and can be used interchangeably with the cosmogram (Ebigbo et al., 2010).

Lastly the Wawa Technique, also derived from Nigerian culture, prompts the client to say Wa (no) to an issue that is against moral order or the well-being of the individual (Ebigbo et al., 1997). It is used to build willpower in clients to refuse something that is against moral order and demonstrates wisdom and good cultural orientation. Proverbs are also used to help to establish moral order and are grounded in cultural, traditional, and religious beliefs (Ebigbo et al., 2017).

The goal of the Wawa Technique is to make the client cognizant and detest the consequences of poor behavior, problematic thinking, or depression that is coupled by external pressures. It can also be used in combination with a behavior chart to monitor adherence in treatment (Ebigbo et al., 2012).

### Expressive Arts

Two studies identified interventions that included artistic expression as best practices for counseling West African children. Narration and storytelling are recognized in the qualitative study by Asagba (2015) using Long's logotherapeutic intervention 3-step model. The storytelling aspect of this model provides hope and promise to sexual assault victims through the identification of rebirth using symbols such as butterflies. This article also provides other expressive art therapeutic forms such as Frankls Mountain, which identifies the challenges they have come to face due to their assault and encourages forgiveness as a tool to heal (Asagba, 2015).

A study by Stepakoff (2016), evaluates trauma-informed practices for those West African survivors of torture and war. The stage-model of trauma and recovery includes a wide range of affect-laden experiences that encourage expressive arts as a technique to healing. Use of symbolism in forms of drawing, drama, song, and other forms of communicative habits encourages resilience and hope. Children who have endured traumatic experiences through torture such as mutilation and witnessing of killings, use these therapeutic methods to provide them a voice to speak without consequences. The countenance of the client requires the rapport to be built with the counselor, and trust established to promote the freedom to process through expression (Stepakoff, 2016).

### Western Psychotherapeutic Approaches

Western approaches to psychological treatment are being used in West Africa. Lo and Dzokoto (2005), suggested that it is important to critically examine the use of western interventions due to their rise in popularity and use. In their examination, Lo and Dzokoto (2005), the use of western style interventions and the appropriate nature of these counseling methods in non-western countries was explored for cultural sensitivity and best practice. A common approach between Ghana and the United States is the use of talk therapy. Both countries use psychiatrists and psychologists. Clients receive treatment based on a mental health diagnosis and can seek both inpatient and outpatient support. However, there are significant cultural differences in the use of western approaches to treatment. In Ghana, the psychologist is perceived to be more of an expert on mental health than a psychiatrist due their perceived ability to better understand human behavior (Lo and Dzokoto, 2005).

The use of mental health counseling is not limited to only psychiatrists and psychologists in Ghana. Similar to western approaches to community counseling, they also have school counselors, religious counselors, and public health educators (Lo and Dzokoto, 2005). Similar to the United States (U.S.), counseling services are becoming more socially acceptable in Ghana. An integrative approach is used to allow services to work together for a preventive approach to mental health. In contrast to the United States, Ghana has remained true to their culture by using traditional healers to support mental health (Lo and Dzokoto, 2005). Although this is a difference in approach, the use of multiculturalism is becoming more important in the U.S. and around the world to provide a more holistic approach to mental health treatment.

In 2005 the Center for Victims of Torture concluded their support and mental health treatment for Liberian and Sierra Leonian survivors of civil war (Stepakoff et al., 2006). The quest was identified as the Guinea project. A stage-oriented model of trauma recovery was used to guide their treatment. Trauma specialist and American psychiatrist, Dr. Judith Herman's trauma approach provided the most influence of the model (Herman, 2015). The three phases of her model are safety and stabilization, processing traumatic memories, and the process of resolution and recovery (Herman, 2015). The treatment team used approaches that are like community and group counseling in western approaches of mental health treatment. The results indicated a decrease in trauma symptoms for participants and an increase of positive daily function and use of social support. The team provided counseling to over 4,000 clients and provided support to at least 15,000 people by delivering supportive services (Stepakoff et al., 2006).

The Guinea team's initial objective was to establish trust and to promote safety. This was initiated by providing the nature and limitations of their treatment. This is parallel to providing consent of treatment. Clients were encouraged to establish ground rules and expectations. Confidentiality and respect were promoted among the group members and the team. Group members were encouraged to get to know about other group members. This initial stage of treatment is very similar to western approaches; however, there are some significant differences that the Guinea team promoted to establish safety among group members. Group members were encouraged to spend time together outside of the group sessions. In addition, group members were not encouraged to share their trauma story at the beginning of treatment. Clients were encouraged to share what their lives were like before they experienced the trauma of war (Stepakoff et al., 2006).

Cognitive therapy is another western approach that was consistently used in this treatment. The team noticed that clients struggled with negative self-talk and made negative statements that were not accurate in accordance to reality. Positive self-talk and positive reframes were encouraged in a cultural manner. The team encouraged group members to practice positive self- talk within the group along with facilitators. They noticed that their approach to positive self-talk represented cultural significance for the clients. Ultimately clients of various ages expressed appreciation for the services provided (Stepakoff et al., 2006).

## Discussion

In reviewing counseling interventions best appropriate for children and adolescents of West African descent, exploration of best practices is still a necessity. In the five articles included in this manuscript, children and adolescents' counseling interventions were identified for treatment of various mental health illness specific to West African culture and experiences.

While examining existing data on counseling techniques used with children, research identifying traditional healers and spiritual practices were reported to be used to cast out demonic behaviors or witchcraft (Yoder et al., 2016). The presence of the traditional healers is limited in the literature and does not elaborate on their role as a holistic healer for mental health. When discussing spirituality, both the Ebigbo et al. (2017) article and the Lo and Dzokoto (2005) article highlighted the presence of a spiritual leader such as a priest, Ideal Master or traditional healer to help the client with stress and anxiety reduction, and prayer to assist in reducing the experiences of trauma. The Ideal Master is also mentioned by Lo and Dzokoto (2005) regarding cultural related interventions. The Lo and Dzokoto (2005) article discusses the inclusion of cultural values in delivering spiritual and western approaches and interventions to clients. Increased knowledge of culture and its life application for the client increases processing of problems and conceptualizations with more context and accuracy (Lo and Dzokoto, 2005). During the review of the Ideal Masters as a cultural intervention tool, other cultural interventions such as Harmony Restoration Therapy, Meseron Treatment, cosmograms, and the Wawa Technique are also identified in the Ebigbo et al. (2017) article. These interventions align with elements of West African culture. Cultural interventions that include the value of relationships, symbols, and language create opportunities to address adversities in a connecting experience that emphasizes shared practices and belief. For expressive arts, two articles found produced interventions that use oral communication and symbolism for healing. Counselors working with clients who have experienced traumatic experiences such as sexual abuse or physical abuse may use expressive arts in their sessions. Research from older journals avoid discussion of traditional techniques to therapy due to holistic beliefs regarding healing. Techniques that incorporate holistic practices through creative skill and application are neglected such as expressive arts (Bulus, 1989). Asagba (2015) discusses the use of storytelling through symbolism to identify challenges and encourage forgiveness and rebirth, and Stepakoff et al. (2006) uses symbolism in the form of song, drawings and other methods as a means of expression to lead toward healing—thus, there is a common thread between these two sources. Lastly, Western psychotherapeutic approaches were presented throughout the articles of Lo and Dzokoto (2005), Stepakoff et al. (2006), and Herman (2015) in an integrated approach to understanding the experience of West African families and children. Interventions delivered to West African communities such as stage-oriented model of recovery, trauma recovery, and group counseling incorporated culture appropriation. Health professionals such as psychologists, traditional healers, religious and school counselors, and health educators are encouraged to use this approach.

There is growing research and literature on western approaches to mental health in West Africa; however, there is limited research concerning specific interventions and experiences with children. Findings on counseling practices were more prevalent in other parts of the continent such as South Africa (Myers et al., 2019); therefore, review of practices in West Africa was deemed to be important for this manuscript. The articles presented a community counseling approach to treatment. Talk therapy, group counseling, consent, confidentiality, cognitive therapy, and multiculturalism, are all themes that were identified that are like western styles of treatment. However, culture and spirituality are the primary differences that separate how treatment is uniquely presented and received in West Africa. In reference to culture, human behavior is valued at a high level; therefore, the psychologist is viewed as more of an expert than is a psychiatrist, who may offer a more medically based approach. In reference to spirituality, traditional healers are still viewed as a source of support for mental health.

## Conclusion

Political instability, war violence, terrorism, chronic illness, and abuse are themes that were identified in the reviewed articles as adverse child experiences that place children at a high risk for mental health needs in West Africa. The trauma associated with these situations is treated from a mixture of spiritual, cultural, expressive arts, and western style approaches that are related to the culture of the region.

## Implications for Future Research

Mental health awareness and the need for support of children concerning mental health are growing in West Africa. A gap in research that was revealed in the review of these articles was the lack of psychotherapeutic interventions designed specifically for children. The research suggested that children are being exposed to adverse childhood experiences and severe trauma; however, there does not appear to be a universal approach or agreement regarding best practices for children. This gap in research can serve as an opportunity to further explore specific interventions tailored to the needs of children in West Africa and to establish best practices for mental health interventions in the region.

## Author Contributions

SC completed abstract, designed the model and the computational framework, and analyzed the data. SC, CJ, and QS wrote the manuscript and carried out the implementation of the search and study.

## Conflict of Interest

The authors declare that the research was conducted in the absence of any commercial or financial relationships that could be construed as a potential conflict of interest.
